# Supplementary material for: Metabolic network percolation quantifies biosynthetic capabilities across the human oral microbiome
Source: eLife. 2019 Jun 13;8:e39733. doi: 10.7554/eLife.39733 (PMC6609349; doi:10.7554/eLife.39733)
Supplement: Transparent reporting form [file elife-39733-transrepform.pdf]

eLIFE
1st Floor
24 Hills Road
Cambridge CB2 1JP, UK
P 01223 855340
W elifesciences.org
T @elife

eLife's transparent reporting form

We encourage authors to provide detailed information *within their submission* to facilitate the interpretation and replication of experiments. Authors can upload supporting documentation to indicate the use of appropriate reporting guidelines for health-related research (see EQUATOR Network), life science research (see the BioSharing Information Resource), or the ARRIVE guidelines for reporting work involving animal research. Where applicable, authors should refer to any relevant reporting standards documents in this form.

If you have any questions, please consult our Journal Policies and/or contact us: editorial@elifesciences.org.

Sample-size estimation

- You should state whether an appropriate sample size was computed when the study was being designed
- You should state the statistical method of sample size computation and any required assumptions
- If no explicit power analysis was used, you should describe how you decided what sample (replicate) size (number) to use

Please outline where this information can be found within the submission (e.g., sections or figure legends), or explain why this information doesn't apply to your submission:

Our computational analysis utilized random sampling in order to compute the relevant metrics. The default parameters used in our algorithm, including the number of samples and runs, can be found in the methods section "Algorithm functions".

Replicates

- You should report how often each experiment was performed
- You should include a definition of biological versus technical replication
- The data obtained should be provided and sufficient information should be provided to indicate the number of independent biological and/or technical replicates
- If you encountered any outliers, you should describe how these were handled
- Criteria for exclusion/inclusion of data should be clearly stated
- High-throughput sequence data should be uploaded before submission, with a private link for reviewers provided (these are available from both GEO and ArrayExpress)

Please outline where this information can be found within the submission (e.g., sections or figure legends), or explain why this information doesn't apply to your submission:

See above

eLife Sciences Publications, Ltd is a limited liability non-profit non-stock corporation incorporated in the State of Delaware, USA, with company number 5030732, and is registered in the UK with company number FC030576 and branch number BR015634 at the address 1st Floor, 24 Hills Road, Cambridge CB2 1JP | August 2014

Statistical reporting

- Statistical analysis methods should be described and justified
- Raw data should be presented in figures whenever informative to do so (typically when N per group is less than 10)
- For each experiment, you should identify the statistical tests used, exact values of N, definitions of center, methods of multiple test correction, and dispersion and precision measures (e.g., mean, median, SD, SEM, confidence intervals; and, for the major substantive results, a measure of effect size (e.g., Pearson's r, Cohen's d)
- Report exact p-values wherever possible alongside the summary statistics and 95% confidence intervals. These should be reported for all key questions and not only when the p-value is less than 0.05.

Please outline where this information can be found within the submission (e.g., sections or figure legends), or explain why this information doesn't apply to your submission:

Statistical analyses using linear modeling were performed for the results sections "Large-scale analysis of biosynthetic capabilities across the human oral microboime", and "Capturing specific biosynthetic patterns across human oral microbiome organisms". Details regarding statistical methods including alpha-values and multiple hypothesis corrections are included in the corresponding methods sections. Additional statistical analyses using Spearman's rank correlation and Mantel permutation test were performed for the results sections "Using the E. coli core metabolic network to demonstrate features of metabolite producibility", "Metabolite producibility points to putative metabolic mechanisms for E. coli auxotroph co-cultures", "Metabolic similarity correlates with microbial co-occurrence in the human oral microbiome", and "Biosynthetic properties predicted in a cluster of fastidious human oral microbiome organisms". All relevant p-values have been reported in the text or supplementary figures/tables, and all methods are described in detail in the corresponding methods sections.

(For large datasets, or papers with a very large number of statistical tests, you may upload a single table file with tests, Ns, etc., with reference to sections in the manuscript.)

Group allocation

- Indicate how samples were allocated into experimental groups (in the case of clinical studies, please specify allocation to treatment method); if randomization was used, please also state if restricted randomization was applied
- Indicate if masking was used during group allocation, data collection and/or data analysis

Please outline where this information can be found within the submission (e.g., sections or figure legends), or explain why this information doesn't apply to your submission:

Not applicable

Additional data files ("source data")

- We encourage you to upload relevant additional data files, such as numerical data that are represented as a graph in a figure, or as a summary table
- Where provided, these should be in the most useful format, and they can be uploaded as "Source data" files linked to a main figure or table
- Include model definition files including the full list of parameters used
- Include code used for data analysis (e.g., R, MatLab)
- Avoid stating that data files are "available upon request"

Please indicate the figures or tables for which source data files have been provided:

* The main data from our study, presented in Figure 4, is available as an excel table in Supplementary Table 3.
* All additional data and scripts necessary for recreation of all figures of the paper are available on GitHub at https://github.com/segrelab/biosynthetic_network_robustness (Latest commit f82f1e0 on Apr 23).
* Genomic sequences for the TM7 (and host) strains are available on NCBI GenBank, with the following accession numbers: Saccharibacteria (TM7) bacterium HMT-488 strain AC001: NCBI CP040003, Saccharibacteria (TM7) bacterium HMT-955 strain PM004: NCBI CP040008, Pseudopropionibacterium propionicum HMT-439 strain F0700: NCBI CP040007.
